# Paraparesis due to angio-neurotropic *Gurltia paralysans* in a domestic cat (*Felis catus*) and retrospective study on feline gurltiosis cases in South America

**DOI:** 10.3389/fvets.2024.1322819

**Published:** 2024-01-19

**Authors:** Marcelo Gómez, Pamela Muñoz, Manuel Moroni, Marcelo Mieres, Valentina Bernal, Carla Rosenfeld, Anja Taubert, Carlos Hermosilla

**Affiliations:** ^1^Instituto de Farmacología y Morfofisiología, Facultad de Ciencias Veterinarias, Universidad Austral de Chile, Valdivia, Chile; ^2^Instituto de Patología Animal, Facultad de Ciencias Veterinarias, Universidad Austral de Chile, Valdivia, Chile; ^3^Instituto de Ciencias Clínicas Veterinarias, Facultad de Ciencias Veterinarias, Universidad Austral de Chile, Valdivia, Chile; ^4^Instituto de Medicina Preventiva Veterinaria, Facultad de Ciencias Veterinarias, Universidad Austral de Chile, Valdivia, Chile; ^5^Institute of Parasitology, Justus Liebig University Giessen, Giessen, Germany

**Keywords:** *Gurltia paralysans*, feline gurltiosis, neuroparasitosis, domestic cats, paraparesis

## Abstract

**Introduction:**

The nematode *Gurltia paralysans* is a neglected angio-neurotropic parasite causing chronic meningomyelitis in domestic cats (*Felis catus*) as well as wild felids of the genus *Leopardus* in South America. Adult *G. paralysans* nematodes parasitize the leptomeningeal veins of the subarachnoid space and/or meningeal veins of the spinal cord parenchyma. The geographic range of *G. paralysans* encompasses rural and peri-urban regions of Chile, Argentina, Uruguay, Colombia and Brazil.

**Methods:**

This case report presents clinical and pathological findings of a *G. paralysans*-infected cat suffering from severe thrombophlebitis and meningomyelitis resulting in ambulatory paraparesis. Neurological examination of affected cat localized the lesions at the thoracolumbar (T3–L3) and lumbosacral (L4–Cd4) segments. Molecular and morphological characteristics of extracted nematodes from parasitized spinal cord veins confirmed *G. paralysans*. Additionally, data obtained from a questionnaire answered by cat owners of 12 past feline gurltiosis cases (2014–2015) were here analyzed. Questionnaire collected data on age, gender, geographic location, type of food, hunting behavior, type of prey, and other epidemiological features of *G. paralysans*-infected cats.

**Results and Discussion:**

Data revealed that the majority of cats originated from rural settlements thereby showing outdoor life styles with hunting/predatory behaviors, being in close contact to wild life [i.e. gastropods, amphibians, reptiles, rodents, birds, and wild felids (*Leopardus guinia*)] and with minimal veterinary assistance. Overall, this neglected angio-neurotropic *G. paralysans* nematode still represents an important etiology of severe thrombophlebitis and meningomyelitis of domestic cats living in endemic rural areas with high biodiversity of definitive hosts (DH), intermediary (IH), and paratenic hosts (PH). The intention of this study is to generate awareness among veterinary surgeons as well as biologists on this neglected feline neuroparasitosis not only affecting domestic cats but also endangered wild felid species of the genus *Leopardus* within the South American continent.

## 1 Introduction

*Gurltia paralysans* is a neglected and re-emerging nematode placed in the family Angiostrongylidae (superfamily Metastrongyloidea) and the only member so far reported for the genus *Gurltia* (1, 2). *G. paralysans* causes severe meningomyelitis, known as feline gurltiosis, affecting mainly domestic cats (*Felis catus*) and wild felid species of *Leopardus* (2). The parasite has been recorded in various South American countries, including Chile, Argentina, Colombia, Uruguay and Brazil (3–13). Recently, there have been reports of the first case outside of South America in Tenerife (Canary Islands, Spain), and a sporadic anecdotal case in USA previously (14). Adult *G. paralysans* nematodes (female 20–30 mm × 0.1 mm; males 12–15 mm × 0.1 mm) reside in the subarachnoid space, specifically in the thoracic, lumbar and sacral spinal cord segments of affected wild or domestic cats which represent the definitive hosts (DH). *G. paralysans*-induced meningomyelitis causes symptoms of progressive hindlimb weakness, pelvic limb ataxia, tail paralysis, urinary, and fecal incontinence (2–5). The life cycle of *G. paralysans* is still not fully understood, but hypothetically either an infected-mollusk intermediate host (IH) or an infected-bird, −amphibian and/or –reptile, acting as paratenic host (PH), is ingested by a domestic- or a wild cat (2, 6). The infective third-stage larvae (L3) migrate through the mucosal layer of the digestive system to the venous or lymphatic system of the abdominal viscera, and then via veins connections or anastomosis of the azygos or caval venous system with thoracic, lumbar or sacral intervertebral veins to reach the vertebral venous plexus. These vascular connections could also explain the presence of *G. paralysans* eggs and adults in remote anatomic sites, such as the cerebrum, cerebellum and, in more recent reports, in the anterior chamber of the eye (14–19).

Infectious etiologies of feline meningomyelitis include viruses (e.g., PIF, FelV, and FIV), and bacteria (e. g. *Pasteurella multocida*), fungal, protozoal, and nematode agents (e. g. *Toxoplasma gondii*, *Sarcocystis* spp., *Baylisascaris procyonis*, *Dirofilaria immitis*, and *Aelurosrongylus abstrusus*) (2, 20, 21). Consistently, cases of aberrant or ectopic larval migration to central nervous system (CNS) of *B. procyonis*, *D. immitis*, and *A. abstrusus* have been reported and thereby producing spinal cord lesions in domestic cats (22, 23). Nonetheless, *G. paralysans* is an emerging metastrongyloid nematode with a marked angio-neurotropic character of adult nematodes migrating to the veins of spinal subarchnoid space for mating purposes (2, 6).

The objectives of this article were to report on a recent case of chronic paraparesis associated to a domestic cat in Southern Chile and retrospectively analyze epidemiological aspects of 12 previous cases of this uncommon and neglected feline neuroparasitosis.

## 2 Case presentation

A 3 year old, 3.5 kg body weight (BW), female domestic cat was referred in January 2023 to the Veterinary Hospital at the University Austral of Chile (UACH), for chronic and progressive pelvic limb ataxia that had progressed to non-ambulatory paraparesis over a period of 3 months. The cat was kept indoor/outdoor in a rural settlement close to the city Puerto Varas in Southern Chile. There were external abrasions on dorsal metatarsal regions indicating chronic weakness of pelvic limbs. Neurologic examination revealed physiological mental status and cranial nerve activities, non-ambulatory paraparesis, decreased muscle tone of pelvic limbs, decreased flexor reflexes in pelvic limbs, normal patellar reflexes with no alteration in postural and spinal reflexes of forelimbs and tail paralysis. The bladder was full and difficult to express but there was no defecation disorder. Spinal hyperesthesia was detected in response to palpation of lumbar and lumbosacral regions. The neurological examination indicated that neuroanatomical lesion localization was at the T3–L3 and L4–Cd4 spinal cord segments. Complete blood count, biochemistry panel, fecal examination, spinal radiographs were unremarkable. Differential diagnosis included spinal trauma, infectious agents (i.e. viral, bacterial, fungal, protozoa, and nematodes), non-infectious inflammatory disorders (i.e. meningomyelitis of unknown origin [MUO]) and neoplastic etiologies (i.e. lymphoma and meningioma). Due to owner request, the cat was euthanized and *post mortem* examination was performed.

**Figure 1:**
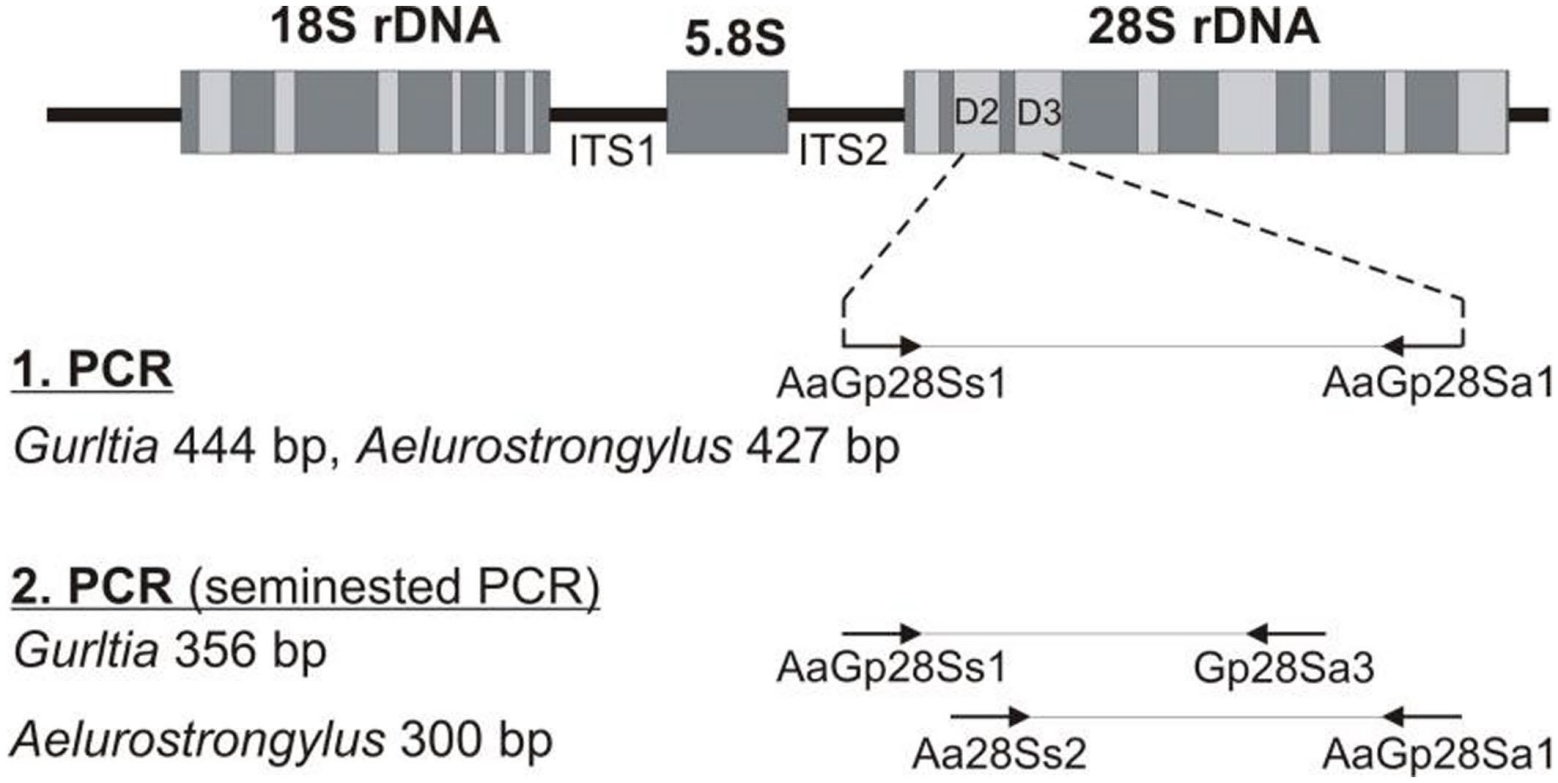
Schematic representation of the seminested PCR technique used for the *in vivo* diagnosis of *G. paralysans-* and *Aelurostrongylus abstrusus* infections.

In order to detect *Gurltia* DNA, polymerase chain reaction (PCR) targeting the 28S rDNA was performed. A serum sample was obtained for a semi-nested PCR analysis for *G. paralysans* DNA (U2 universal oligonucleotide and *G. paralysans*-specific oligonucleotide E1: Gp28Sa3) and *Aelurostrongylus abstrusus* (U1 universal oligonucleotide and *A. abstrusus*-specific E1: Aa28Ss2) based on previously reported studies (1) (Figure 1). DNA extraction from the serum sample was performed according to the manufacturer instructions, using an E.Z.N.A.^®^ Tissue DNA Kit D3396-02 (Omega Bio-tek, Inc., Norcross, GA, United States). The first PCR amplified DNA from both parasite species using universal oligonucleotides (forward: AaGp28Ss1 5′-CGAGTRATATGTATGCCATT-3′, reverse: AaGp28Sa1 5′- AGGCATAGTTCACCATCT-3′) based on identical conserved sequences. The second (semi-nested) specific PCR differentiated *G. paralysans* (universal forward primer AaGp28Ss1, *Gurltia* reverse primer Gp28Sa3 5′-TCTTGCCGCCATTATAGTAG-3′) from *Aelurostrongylus abstrusus* DNA (*Aelurostrongylus*-specific forward primer Aa28Ss2 5′-CGTTGATGTTGATGAGTATC-3′, universal reverse primer AaGp28Sa1). Reaction conditions for the first PCR were as follows: 5 min initial denaturation at 94°C 35 to 40 cycles of 30 s at 94°C, 30 s at 54°C and 30 s 72°C extension, followed by a 5 min 72°C elongation. From first PCR, 1 μL of the reaction was used as template for the second semi-nested PCRs under the following conditions: 94°C for 1 min, 35 cycles of 30 s at 94°C, 30 s at 56°C, 30 s at 72°C and a final elongation for 5 min 72°C. PCR products were analyzed by agarose gel electrophoresis (2%). Sequencing of the amplicon was performed in an automated DNA analyzer (Applied Biosystems). Amplification products were run on 2.5% ethidium bromide agarose gels and visualized under ultraviolet light.

### 2.1 Data collection

Additionally, data obtained from a questionnaire answered by cat owners of 12 past feline gurltiosis cases (2014–2015) were analyzed (Tables 1, 2 and 3). Questionnaire data included demographic- and risk factors using face-to-face interviews with cat owners. Data collected included age, gender, types of food, hunting behavior, type of prey, lifestyle, presence of contacting wildlife animals and duration of clinical signs. The food type included the categories of canned food, dry food and homemade food. The lifestyle categories were “indoor housing”, “outdoor housing”, “access to the outdoors” and “outdoor hunting”.

**Table 1:** Variable demographic description collected from cat owners of 12 past feline gurltiosis cases in Southern Chile.

Variable	*n*°	%
**Age**		
<1 y	1	8.3
>3 y	7	58.3
ND	4	33.3
Total	12	100.0
**Breed**		
DSH	5	41.7
DLH	3	25.0
ND	4	33.3
Total	12	100.0
**Sex**		
Female	6	50.0
Male	6	50.0
Total	12	100.0
**Spayed/castrated**		
No	10	83.3
Yes	2	16.7
Total	12	100.0

DSH, domestic short hair cat; DLH, domestic long hair cat; ND, non data available.

**Table 2:** Variable demographic information collected from cat owners of 12 past feline gurltiosis cases in Southern Chile.

Veterinary care	*n*°	%
No	9	75.0
Yes	3	25.0
Total	12	100.0
**Vaccination/anthelmintic status**		
Updated	1	8.3
Sometimes	3	25.0
Never	8	66.7
Total	12	100

**Table 3:** Environmental and feeding variables reported from cat owners of 12 past feline gurltiosis cases in Southern Chile.

Variable	Cases											
	1	2	3	4	5	6	7	8	9	10	11	12
**Habitat**												
Cat goes outside	✓	✓	✓	✓	✓	✓	✓	✓	✓	✓	✓	✓
Cat lives outdoor	no	✓	✓	no	✓	✓	✓	✓	✓	✓	✓	✓
Cat interacts with other outdoor cats	✓	no	✓	no	✓	no	✓	✓	✓	✓	✓	✓
**Environment**												
Proximity to native forest	✓	✓	✓	no	✓	✓	✓	✓	✓	✓	✓	✓
**Food**												
Processed cat food	✓	✓	✓	✓	✓	✓	✓	✓	✓	✓	✓	✓
Home-made food	✓	–	–	–	✓	✓	✓	✓	–	–	–	–
Milk	–	–	–	–	–	–	–	–	–	–	✓	✓
Hunting	✓	✓	✓	✓	✓	✓	✓	✓	✓	✓	✓	✓
**Type of prey**												
Birds	✓	nd	✓	nd	✓	✓	✓	✓	✓	✓	✓	✓
Insects	nd	nd	nd	✓	✓	✓	nd	nd	nd	nd	✓	✓
Rodents	✓	✓	✓	nd	✓	✓	✓	✓	✓	✓	✓	✓
Small lizards	✓	nd	✓	nd	✓	✓	nd	nd	nd	✓	✓	✓
**Presence of wild animals**												
Wild felids (*Leopardus guigna*)	✓	–	–	–	✓	–	✓	✓	✓	✓	✓	✓
Frogs	–	–	✓	–	✓	–	–	–	–	–	✓	✓
Foxes (*Pseudalopex griseus, Pseudolopex culpaeus*)	–	✓	–	–	–	–	–	–	–	–	–	–
Puma (*Puma concolor*)	–	✓	–	–	–	–	–	–	–	–	–	–
Others (small lizards, birds, and rodents)	✓	✓	✓	✓	✓	✓	✓	✓	✓	✓	✓	✓

nd, non data available.

## 3 Results

**Figure 2:**
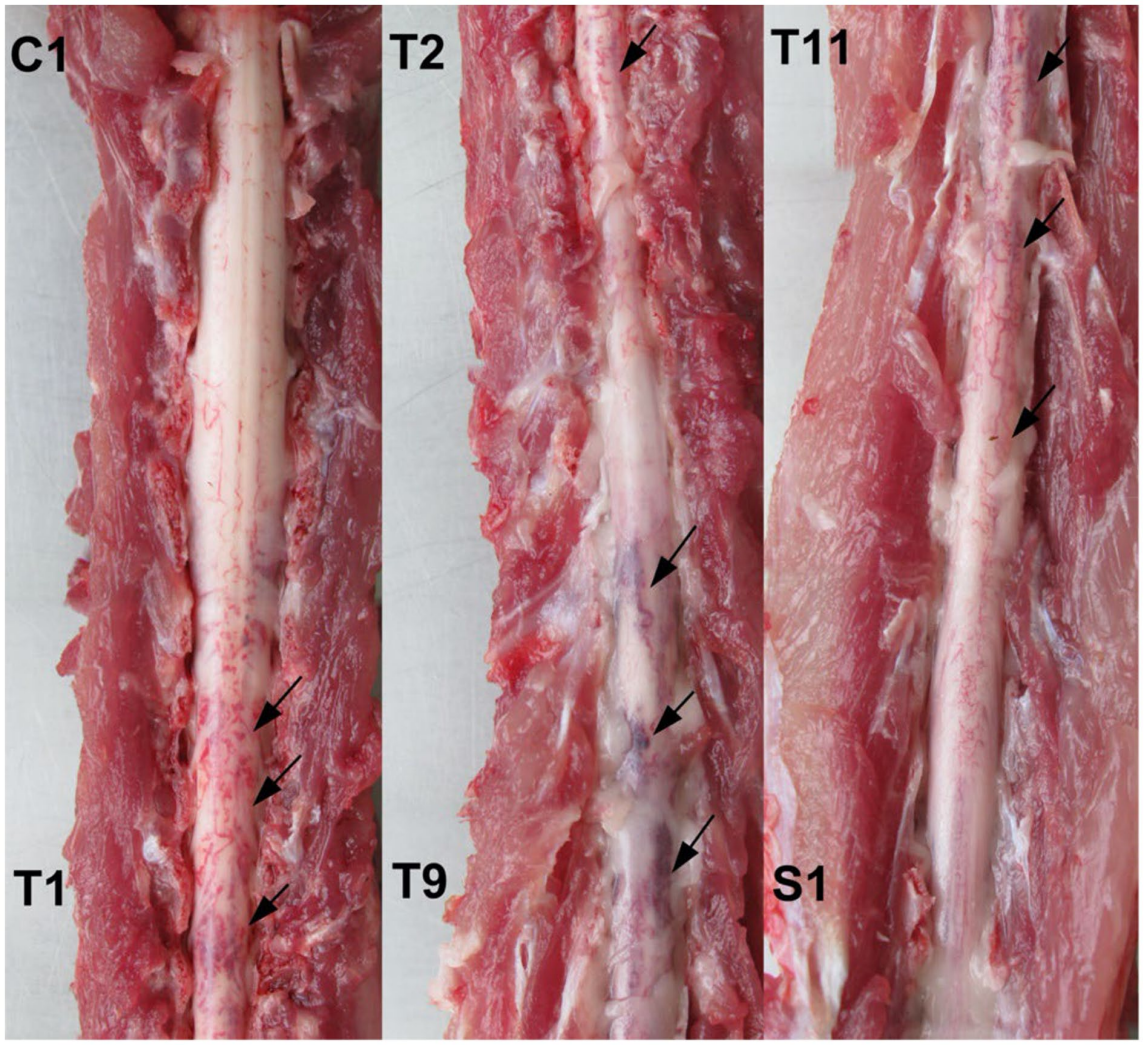
*Post mortem* spinal cord specimen from an affected domestic cat with *G. paralysans* infection. Multiple areas of submeningeal congestion (arrows) are observed in the cervicothoracic-, thoracic-, and lumbar spinal cord segments.

**Figure 3:**
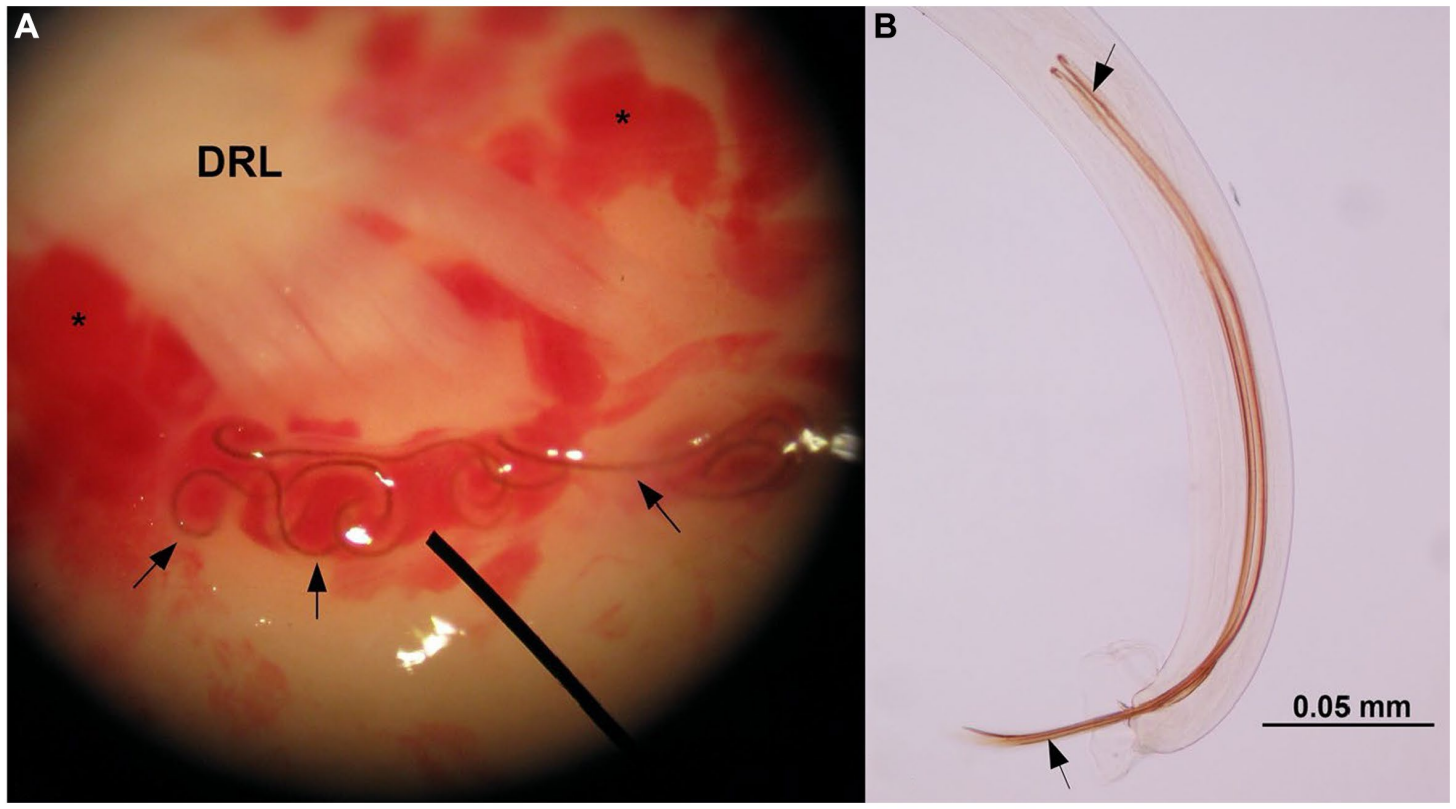
Stereoscopic microscope illustration indicating the presence of adult nematodes of *G. paralysans* (arrows) in a vein at the base of the dorsal rootlets (DRL) of a lumbar spinal nerve. Multiple areas of congestive leptomeningeal vessels are observed (asterisks). Video is available in the Supplementary material **(A)**. Caudal end of an adult male specimen of *G. paralysans* indicating the spicules (arrows).

**Figure 4:**
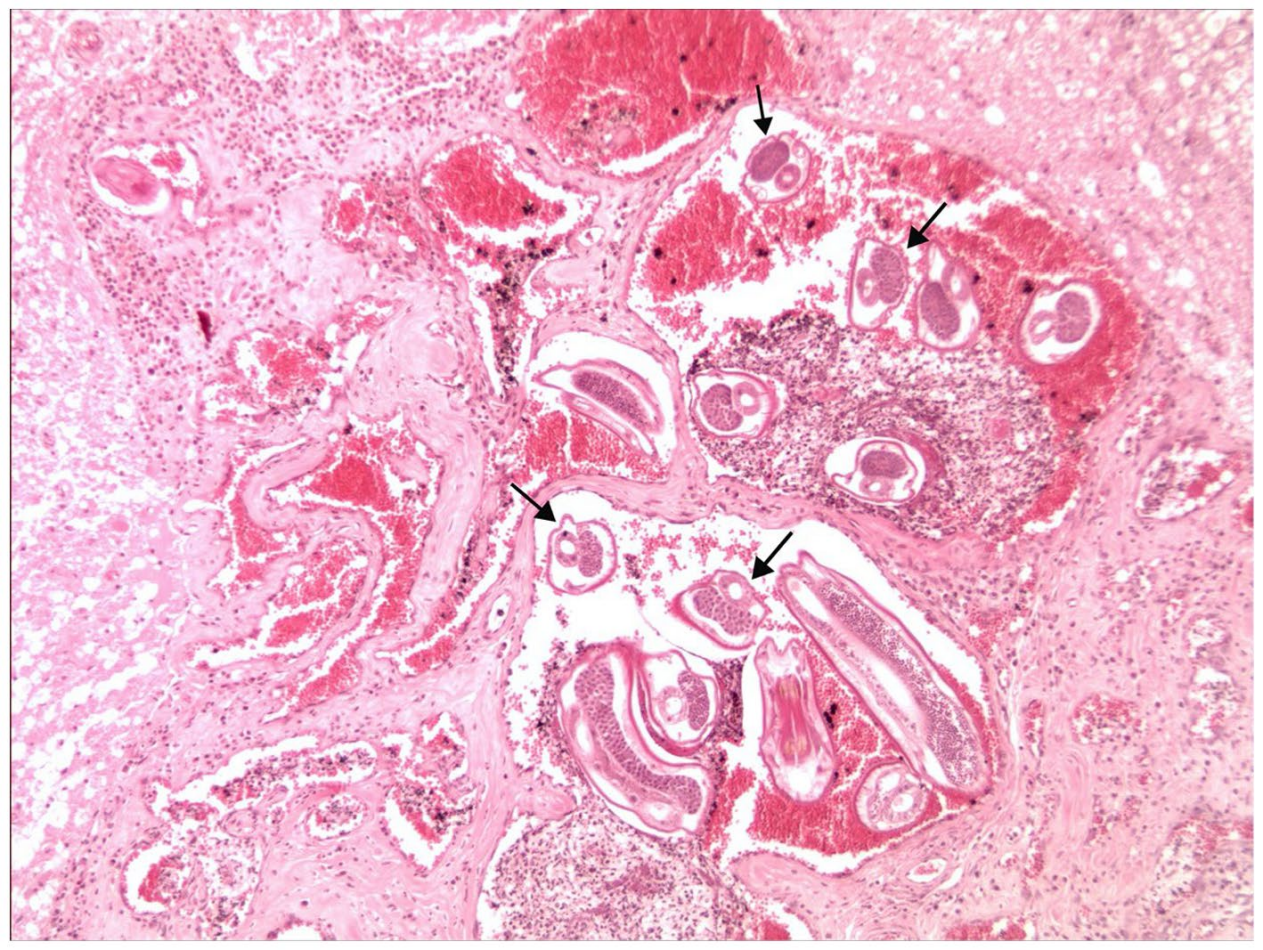
Histopathological sections of the lumbar spinal cord parenchyma of the affected cat. Several adult *G. paralysans* specimen cross section (arrows) are observed within the spinal cord vasculature and surrounded by blood and inflammatory cells. H&E. Original magnification 100X.

Results obtained from the molecular analysis confirmed identification of *G. paralysans* (GenBank: J975484) but were negative for *A. abstrusus*. On macroscopic *post mortem* evaluation, no significant lesions were observed in the brain up to C8 spinal cord segment. Submeningeal congestive vessels, i.e. indicative of progressive thrombophlebitis, were observed at the cervicothoracic, mid thoracic and lumbar spinal cord segments (Figure 2). Moving adult nematodes were observed in the leptomeningeal vasculature near the dorsal nerve rootlets of the lumbar segment by examination under a dissecting miscroscope and then carefully extracted “... (Figure 3A). One of the extracted specimens was approximately 13 mm in length and morphological characteristics were consistent with a male nematode of *G. paralysans* (Figure 3B). Brain and spinal cord samples from affected domestic cat were fixed in neutral buffered 10% formaldehyde. Samples were embedded in paraffin wax, and sections (4 μm) were stained with hematoxylin and eosin (HE). Histopathologic evaluation of the formalin-fixed section of spinal cord revealed meningomyelitis extending the cervicothoracic to the sacral regions. Lesions included congestion of spinal cord and leptomeningeal vasculature and presence of thrombi in the subarachnoid veins and sections of adult specimens of *G. paralysans* in the spinal cord parenchyma (Figure 4).

In the descriptive analysis of the 12 previous cases of feline gurltiosis, variables such as “age > 3 years” and/or “not castrated” were more frequent observed in affected cats (Table 1). The absence of veterinary care and infrequently or never given anthelmintic treatments were more likely observed in affected than regularly treated cats (Table 2). Additionally, cats living “rural” and with “outdoor activities” were more frequently affected by this neuroparasitosis (Table 3).

## 4 Discussion

The feline patient described in this report presented chronic progressive ambulatory paraparesis. The most frequent clinical manifestation of neurological feline gurltiosis is chronic and progressive ambulatory pelvic limb weakness that may end in paraplegia (3–5, 9, 11). Regarding the length of clinical signs, our retrospective study found that the majority of cases (5/12) had a chronic duration (> 1 month) in agreement with other reports. In line, previous studies have shown that the duration of clinical signs can range from 2 weeks to 48 months (5, 9–11, 24). Other signs observed and that have been reported include pelvic limb ataxia, pelvic limb proprioceptive deficit, pelvic limb tremor, pelvic limb muscle atrophy, tail tremors, tail atony, and fecal/urinary incontinence (3–5, 8, 11, 16, 17). Neurological signs had correlative neuroanatomical lesions observed at necropsy and in histopathological samples (2, 5, 24). Haematological abnormalities associated with feline gurltiosis included non-regenerative anaemia and low mean corpuscular haemoglobin concentrations (hypochromia) (2, 5) revealing chronic inflammatory disease or chronic blood loss (2, 5). The eosinophilia associated with parasitic infections has commonly been reported in domestic animals, but is not a frequently observed feature in domestic cats with feline gurltiosis (5), which has also been reported in dogs with neural angiostrongylosis (25, 26). No clinical signs of coagulopathy have been observed in naturally *G. paralysans*-infected cats. However, high levels of urea in the blood (uremia) have been reported probably arising from neurogenic urinary dysfunction (5). A bronchial lavage analysis of five naturally *G. paralysans*-infected cats showed absence of larval stages and eggs (27). Ocular lesions including uveitis, chorioretinitis, posterior synechiae, and corneal oedema, have recently been reported to be associated with the presence of a motile adult specimen of *G. paralysans* in the anterior chamber of the eye of a domestic cat (14).

**Figure 5:**
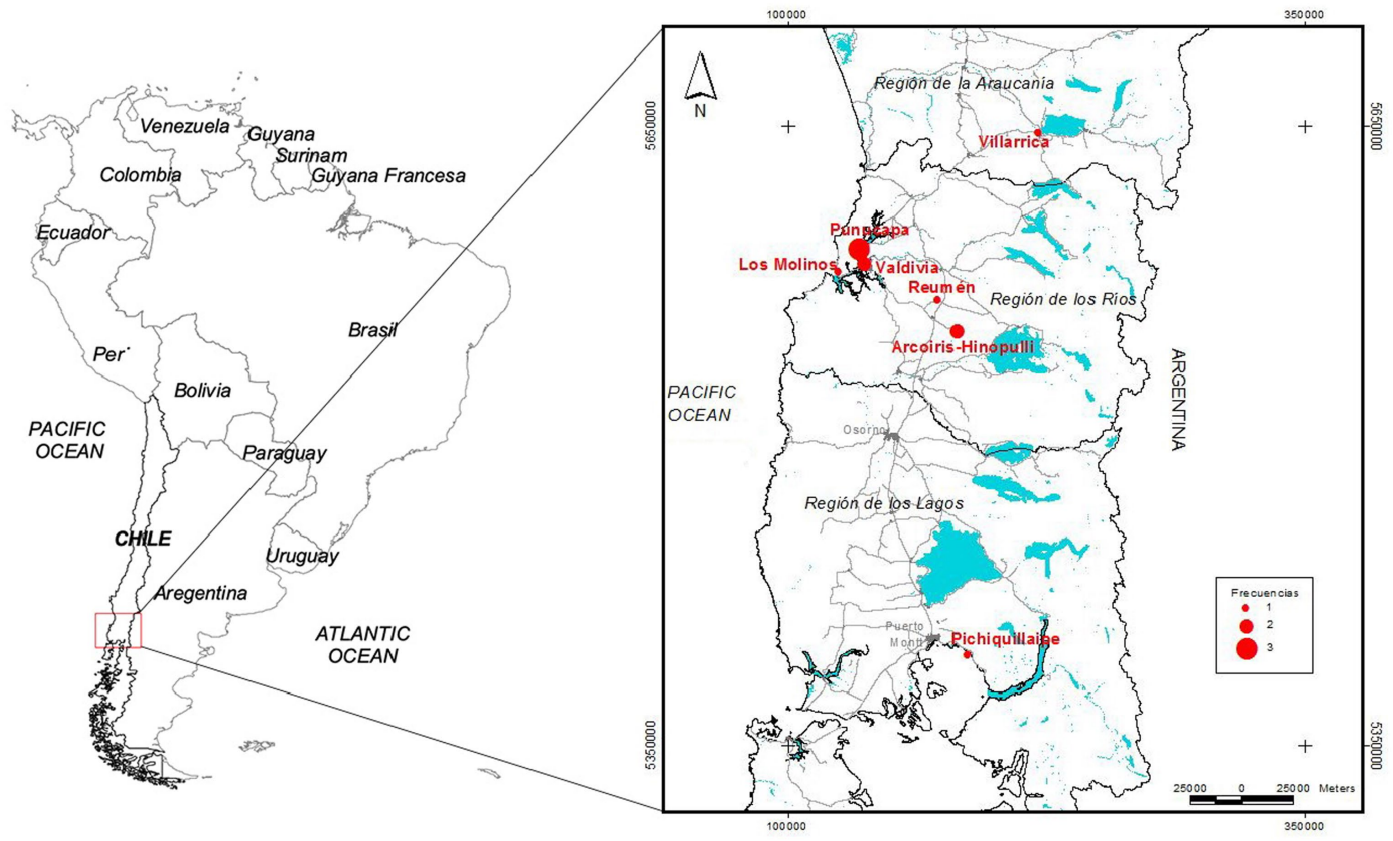
Geographic distribution of the 12 cases of feline gurltiosis, frequencies and wetland locations in Southern Chile.

In line, necropsy findings of *G. paralysans*-infected domestic cat reported in this study, included presence of adult females and males in meningeal veins thereby inducing diffuse leptomeningeal congestion of the lumbar, sacral, and caudal spinal cord segments (Figure 5). These findings are in line with pathological lesions observed in other previous studies (3, 4). Numerous intravascular eggs, nematode larvae and pre-adult stages can be identified histologically in the meningeal veins of the spinal cord, associated with vascular congestion, thrombosis, and thickening of the subarachnoidal vessels (3–5, 11). Presence of mild smooth-muscle hypertrophy, moderate adventitial fibroplasia, and marked subintimal fibrosis of the spinal cord venules (phlebosclerosis) have been reported as well (11). In some specimens, concentric thickening of the venule wall may produce stenosis of the vessel lumen (11). Sections of normal or dilated and tortuous varicose venules may contain thrombi with various levels of organization (11). The spinal cord parenchyma may show areas of malacia, microcavitation, multiple haemorrhages, extensive areas of malacia, with gitter cells and adjacent reactive gliosis and foci of mineralization (4, 11, 24). Lymphocytic infiltrate, intermingled with fewer macrophages, primarily infiltrate the subarachnoid space, forming a perivascular pattern. Mature eosinophils distributed randomly within the leptomeninges have also been observed, which are consistent with broad spinal leptomeningitis and thrombophlebitis (4, 11). Some animals may also show granulomatous leptomeningitis or suppurative leptomeningitis (11). Expression of GFAP, CNPase, factor VIII, CD3 and CD45 in affected spinal cord segments are suggestive of reactive gliosis and chronic inflammatory spinal lesions consequent to the ischemia caused by *G. paralysans-*mediated vascular injury (28). However, no cases of feline gurltiosis have been observed with clinical encephalic syndromes.

The morphological features of the specimen recovered from the affected spinal cord were consistent with an adult male of *G. paralysans* (Figure 3). This identification was further confirmed by a semi-nested PCR showing the expected amplification products and matching with *G. paralysans* sequence.

Historically, the first cases of feline gurltiosis were reported in 1933 in domestic cats from temperate rainforest areas around Valdivia in Southern Chile (29, 30). Subsequently, cases have been reported in other regions of Chile including the IX (i.e. La Araucanía)- and in the X (i.e. Los Lagos) region (2–4, 31) (Figure 4). Since then, other cases have been diagnosed in Argentina (8, 10), Uruguay (9), Colombia (7), and Brazil (11, 24, 25) (Table 4). In Chile, cases of feline gurltiosis have been predominantly reported in rural areas, but understanding of the spatial distribution in this environment is limited (3–5, 19, 29, 30). As above stated, feline gurltiosis has been diagnosed in three different rural regions of Southern Chile: (i) the Araucanía region (e.g., Lastarria), (ii) the Los Rios region (e.g., Punucapa, Niebla, Paillaco, and Futrono), and (iii) the Los Lagos region (e.g., Futrono, Pichirropulli, and Ancud) (4) and more recently in the cities of Puerto Montt, Valdivia, and Temuco (31). In Colombia, six cases of feline gurltiosis in domestic cats were diagnosed in Antioquia (municipalities Tarso and Amagá), with signs of spinal hyperesthesia and paraparesis (7). In Argentina, *G. paralysans* was reported in one cat in the Baradero area of Buenos Aires province, and in three cases in rural areas of the Santa Fé province, specifically in the districts of Las Colonias, San Cristóbal, and Castellanos (8). In Uruguay, between 2008 and 2009, two cases of feline parasitic meningomyelitis caused by *G. paralysans* were documented in the rural region of Fray Bentos (9). Gurltiosis has been identified in Brazil since the mid-1990s, and is known locally as “bambeira,” “derrengado,” or “renga,” which mean wobbly or lame. In 2013, there were four reported cases of cats infected with *G. paralysans* in the state of Rio Grande do Sul (11), and eleven cases were later found in Pernambuco (municipalities of Caetés and Capoeiras), Northeastern Brazil (24). More recently, a 2-year-old cat in São Paulo state in Southeastern Brazil was diagnosed with feline gurltiosis, indicating a wide distribution of the parasite throughout Brazilian territory (25). Additionally, two wild cats, specifically *Leopardus wieddii* and *Leopardus triginus*, were discovered in Parana (Chapecó), showing typical severe spinal cord lesions associated with feline gurltiosis (32, 33). The first case outside of America was documented in Tenerife, Canary Islands, Spain involving a parasitic ocular infection by *G. paralysans* (14). In 1993, a cat in the United States of America (USA) exhibited neurological signs and necropsy findings in the lumbar spine consistent with feline gurltiosis (34). The occurrence of feline gurltiosis, in both Spain and in USA, may have been caused by the introduction of *G. paralysans*-infected domestic cats from South American endemic areas, or by the importation of infected IH or PH, similar to the closely related *Angiostrongylus cantonensis* to the Canary Islands of Spain (35). However, further research is needed for defenitive conclusions.

**Table 4:** Profile of feline *G. paralysans*-induced meningomyelitis and ocular cases including age, geographic area, spinal/ophthalmic location, imaging systems and diagnosis published since 2010.

Author	Location	Cases	Age	Clinical signs	Imaging	Final Diagnosis	PCR confirmation	Extracted nematodes
Gómez et al. 2010	Chile	3	1–4 y	T3–L3 L4–S3	Myelo CT	Histopathology	NP	2F; 2 M 12F; 2 M 12F; 2 M
Moroni et al. 2011	Chile	3	1–3 y	T3–L3 L4–Cd4	NR	Histopathology	NP	–
Alzate et al. 2011	Colombia	6	6–8 m	T3–L3 L4–Cd4	Rx	Histopathology	NP	–
Guerrero et al. 2011	Argentina	1	2y	T3–L3	NR	Histopathology	NP	–
Rivero et al. 2011	Uruguay	2	ND	T3–L3	NR	Histopathology	NP	–
Mieres et al. 2013	Chile	9	8 m–10y	T3–L3 L4–Cd4	Myelo CT, MRI	Histopathology	NP	–
Togni et al. 2013	Brazil	4	ND	T3–L3 L4–S3	NR	Histopathology	NP	–
Bono et al. 2016	Argentina	3	ND	T3–L3	NR	Histopathology	NP	–
Moroni et al. 2017	Chile	1	8 m	T3–L3	Myelo CT	Histopathology	NP	11F; 1 M
Udiz-Rodriguez et al. 2018	Spain	1	2y	Anterior chamber of the eye	NR	Microsurgical extraction of parasites	Yes	1 M
Melo-Neto et al. 2018	Brazil	11	ND	T3–L3	NR	Histopathology	Yes (9/11)	–
Gómez et al. 2020	Chile	10	4 m–36 m	T3–L3 L4–S3	NP	Histopathology Angio Detect TM IDEXX	NP	10F, 2 M
Gutiérrez et al. 2020	Chile	1	4y	L4–S3	NR	NP	Yes	–
Mello Emboaba et al. 2023	Brazil	1	2y	T3–L3	CT	Histopathology	No	–

Myelo CT, computed tomography-myelography; MRI, magnetic resonance imaging; Rx, conventional radiography; ND, non data available; NP, not performed; F, female; M, male.

The epizootiology of the disease has been linked with rural and rainforest areas, in both temperate and tropical humid ecosystems with abundant vegetation (2, 3, 6, 9, 11, 24, 29, 30). In our retrospective study, all 12 cases of feline gurltiosis were from rural areas. The prevalence of *G. paralysans* in South America is unknown, but is likely to be underestimated and the disease under-diagnosed as recently demonstrated (1, 31). Modeling studies indicate that certain regions in Southern Chile and Argentina, as well as areas in Brazil, Uruguay, and Colombia, are at high risk of the spread of metastrongyloid nematodes based on their climatic suitability (2, 36). Feline gurltiosis has no seasonal occurrence pattern and can be detected throughout the year (3, 4, 9). Regarding the presence of wildlife near the habitat of *G. paralysans*-infected domestic cats, owners indicated the presence of terrestrial gastropods (IH), wild felid species guiña (*Leopardus guigna,* DH), small lizards, frogs, snails/slugs and birds (PH). In terms of predation, owners declared that birds, rodents, lizards and insects were the main prey of hunting. These prey species have previously been mentioned as possible PH in the epizootiology of feline gurltiosis (2, 6). Contact with synanthropic animals, such as birds and rodents, might increase the susceptibility to new parasitic infections as reported elsewhere (37, 38). Additionally, in our study high frequency of infected cats showed an outdoor behavior mentioned by the owners, indicating increase exposure or opportunity to ingest *G. paralysans*-infected IH or PH. These results support the fact that outdoor lifestyle by cats increases the risk of infection. The high rainfall that occurs in Southern Chile and the abundant vegetation and large hydrographic areas (rivers, lakes) support the presence of possible IH or PH that could facilitate transmission from wild guiñas (*L. guigna*) to domestic cats mainly in rural areas.

In one report, domestic cats with feline gurltiosis showed co-infections with *A. abstrusus*, although none of them showing respiratory signs (5). This might suggest that *G. paralysans* and *A. abstrusus* may share the same IH and/or PH (2). Factors affecting the distribution of gastropod species are important in determining whether the life cycle of *G. paralysans* can be completed and whether there is a potential contact with suitable DH and/or PH. Previous epizootiological studies have indicated that the endemic range of closely related *Angiostrongylus vasorum* has expanded into new countries and regions (39–41) and might be extrapolated for *G. paralysans*. Models have been used to predict the distribution of *A. vasorum* and the risk of infection based on climatic variables and their effects on the survival rates of infected IH (40). Similar modeling information is required for *G. paralysans* to predict the real distribution range in South American countries with similar climate conditions, high biodiversity of IH, PH and DH. As such, the causes of apparent re-emergence of metastrongyloid parasitoses in domestic animals are still unknown, but several factors may explain the recent increases in reports of feline gurltiosis in several countries (24, 25). Factors such as global warming, changes in the population dynamics of IH and PH, and movement of animals (DH) may explain the increase in reports of feline gurltiosis (39, 40). A recent large scale serological/molecular investigation in Southern Chile, including 171 examined animals, revealed a 54.4% occurrence of feline gurltiosis in domestic cats (31). Nonetheless, specific local and/or global geographic criteria studies, or prevalence studies on wild felids are necessary to better understand their role in transmission. Finally, epidemiological investigations based on Geographic Information System (GIS) considering climatic factors, vegetation indices, humidity, temperature, altitude and biodiversity of DH, IH, and PH are urgently needed to comprehend not only the establishment but also the spread of feline gurltiosis into previously non-endemic regions.

Although questionnaires have obvious limitations, they may be useful for further investigations of re-emerging diseases and for raising awareness among clinicians and owners on neglected feline gurltiosis. Critical risk factors for cats, such as access to wildlife environments with high biodiversity (DH, IH, and PH) and hunting habits, may increase the exposure to *G. paralysans* and therefore be considered in future questionnaire-related studies.

Treatment of feline gurltiosis relays on several anthelmintic drugs including macrocyclic lactones such as ivermectin, moxidectin, selamectin and/or milbemycin (2). The usual dose of ivermectin is 300–400 mg/kg/BW, three times weekly associated with corticosteroids (e.g., prednisolone). Additionally, benzimidazoles such as fenbendazole or ricobendazole have been mentioned as effective anthelmintic drugs in literature (2, 8). However, anthelmintic treatments have been beneficial only in mild or moderate cases of suggestive feline gurltiosis but not in animals with severe non-ambulatory paraparesis, paraplegia or in cases with substantial neurological deficit.

## 5 Conclusion

We reported a case of parasitic meningomyelitis due to *G. paralysans* infection in a domestic cat based on clinical, molecular, morphological and *post mortem* results. Additionally, results of the descriptive epidemiological analysis of 12 previous cases of feline gurltiosis in this study suggest that infection with *G. paralysans* should be suspected based on clinical signs of chronic paraparesis, epidemiological characteristic of wildlife environment proximity and hunting habits, regional endemicity, typical pathomorphological lesions of spinal cord veins, and isolation and characterization of specimens. This report underlines the clinical and epizootiological aspects of this neglected parasitosis in domestic and wild felids. Further studies are needed to better understand the relationships among possible demographic and environmental factors and infection of *G. paralysans* in domestic cats. Taking into account that *G. paralysans* also infect various endangered wild felids, clinicians, biologists, and ecologists involved in feline conservation programs in South, Central and North America should be aware of this neglected parasite.

## Data availability statement

Information for existing publicly accessible datasets is contained within the article.

## Ethics statement

The animal studies were approved by Comité de Bioética “Uso de Animales en Investigación” Universidad Austral de Chile. The studies were conducted in accordance with the local legislation and institutional requirements. Written informed consent was obtained from the owners for the participation of their animals in this study.

## Author contributions

MG: Conceptualization, Investigation, Validation, Writing – original draft, Writing – review & editing. PM: Conceptualization, Investigation, Methodology, Writing – review & editing. ManM: Writing – review & editing, Investigation, Methodology, Validation. MarM: Formal analysis, Investigation, Methodology, Writing – review & editing. VB: Investigation, Methodology, Validation, Writing – review & editing. CR: Conceptualization, Investigation, Methodology, Resources, Writing – review & editing. AT: Investigation, Methodology, Supervision, Validation, Writing – review & editing, Writing – original draft. CH: Investigation, Methodology, Resources, Validation, Writing – original draft, Writing – review & editing.
